# Trabeculectomy: is releasable suture trabeculectomy a cause of better bleb?

**DOI:** 10.22336/rjo.2021.10

**Published:** 2021

**Authors:** Rayees Ahmad Sofi, Prem Chand Kesarvani, Viney Gupta

**Affiliations:** * Department of Ophthalmology GMC, Anantnag, Jammu and Kashmir, India; ** AIIMS New Delhi, India; *** Dr. Rajendra Prasad Center for Ophthalmic Sciences, AIIMS New Delhi, India

**Keywords:** trabeculectomy, releasable suture, intraocular pressure, bleb

## Abstract

**Purpose:** To compare the outcome of fixed suture trabeculectomy with releasable suture trabeculectomy in terms of IOP control, bleb morphology, complications and need of antiglaucoma medication post-surgery.

**Methods:** This study enlisted 200 cases of open angle glaucoma, whose IOP was uncontrolled despite maximal medication. Trabeculectomy was performed using releasable suture in one group of 100 patients and fixed suture in another group of 100 with mitomycin 0.02% in both groups. The study was randomized, the method being the simple randomization. Fornix based trabeculectomy was done in both groups. Two 10-0 nylon releasable sutures were used at two corners of the rectangular flap and one fixed 10-0 vicryl suture was used in the center of the flap. Two mattress sutures (conjunctiva cornea) were also used. Essentially, all the sutures were removed postoperatively over a period of 2-4 weeks depending upon the level of IOP. Mitomycin c 0.02% was used in both groups.

**Results:** The mean preoperative intraocular pressure was 33 ± 12 mmHg in the single suture group and 39 ± 13 mmHg in the releasable suture group (p). We observed a highly significant reduction of intraocular pressure at all times in both groups compared with the preoperative intraocular pressure (P, 0.0001). There was an obvious difference between the bleb morphology between conventional trabeculectomy and releasable suture trabeculectomy. Blebs in releasable suture trabeculectomy were more diffuse, low lying and presented a more ideal vascularity.

**Conclusion:** Releasable suture trabeculectomy is a far much better technique than conventional trabeculectomy. Results are very good in terms of IOP control, post-operative complications, and bleb morphology. They may possibly have a role in wound modulation thereby achieving an ideal bleb, though more large sample studies need to be done.

## Introduction

Glaucoma is the leading cause of blindness worldwide. It is the second most common cause of blindness all over world. It is first most common cause of irreversible blindness. Trabeculectomy is the most commonly performed major glaucoma surgery across the globe. Trabeculectomy is a very good surgical procedure to lower IOP and it is the most commonly performed glaucoma surgery across the globe. The gold standard in glaucoma surgery remains trabeculectomy [**1**]. Trabeculectomy is a penetrating filtration procedure that effectively reduces IOP by allowing aqueous drainage through a sclerostomy, with full thickness penetration of the anterior chamber, establishing a connection with the subconjunctival spaces [**2**]. Filtration procedures result in a raised segment of conjunctiva at the surgical site, commonly referred to as a bleb. Trabeculectomy is a very good surgical procedure to lower IOP, but it does not lack complications. Several postoperative complications like shallow, flat anterior chamber, hypotony and choroidal detachment have been reported [**3**-**6**].

Combined with releasable sutures, trabeculectomy has become a preferred option to treat uncontrollable glaucoma. Releasable suture trabeculectomy allows the minimizing of the post op complications like shallow AC, complicated cataract, and others by reducing the chances of future complications. Use of releasable sutures in trabeculectomy was introduced by Schaffer [**6**]. The sutures can be manipulated in the postoperative period depending upon the level of IOP and help in reducing the IOP. Earlier laser suturolysis was used to perform laser suture lysis that is accompanied by its own set of complications like conjunctival scars and flat bleb [**7**].

## Purpose

To compare the outcome of fixed suture trabeculectomy with releasable suture trabeculectomy in terms of IOP control, bleb morphology, complications and need of antiglaucoma medication post-surgery.

## Methods

This study enlisted 200 cases of open angle glaucoma (no intraocular surgery of any nature was performed previously), whose IOP was uncontrolled despite maximal medication. Trabeculectomy was performed using releasable sutures in one group of 100 patients and fixed suture in another group of 100, with mitomycin 0.02% in both groups. Equal sample size was taken in both groups. Fornix based trabeculectomy was performed in both groups. Two 10-0 nylon releasable sutures were used at two corners of the rectangular flap, one 10-0 vicryl fixed suture was used in the center of flap. Two mattress sutures (conjunctiva cornea) were used. Essentially, all the sutures were removed postoperatively over a period of 2-4 weeks depending upon the level of IOP, other than one central 10-0 vicryl suture. Mitomycin c 0.02% was used in both groups for one minute. The follow up period was one year. We compared the mean pre-operative IOP and the mean post-operative IOP (sum of four IOPS) within a difference of one month. During the first month of follow up, patients were called once a week. In the first month postoperatively, the total four follow ups were on day 7, 14, 21 and 28. This was compared with the analysis of 100 cases of conventional (fixed suture) trabeculectomy with same parameters. The 10-0 nylon sutures at the two corners of the flap in this group were fixed. Ocular massage and needling were performed in both groups wherever necessary and the need was less in the releasable suture group because there was the option of removing the suture. All the surgeries were performed by a single surgeon. All the patients in both groups had to undergo extensive preoperative evaluation like applanation tonometry, gonioscopy and disc evaluation. The study followed the guidelines for glaucoma surgical trials by world glaucoma association. Post-operative, all the patients received antibiotic and steroid combination, mostly moxifloxacin and betamethasone, which was tapered over a period of 4-6 weeks and a cycloplegic agent for one to two weeks. The blebs were classified according to Indiana bleb classification system. Trabeculectomy is a commonly performed procedure in our hospital and permission was granted by the hospital regulatory board. The study abode the tenets laid in the declaration of Helsinki. The Indiana Bleb Appearance Grading Scale (IBAGS) is a simple validated system of assessing and incorporating the four major features of bleb morphology: height, extent, vascularity, and aqueous leak (Seidel test) [**8**].

- Comparison of post op intraocular pressure between the two groups.

- Condition of the bleb, whether diffuse, elevated, or vascularized, was also noted.

- Comparison of few main complications between the two groups.

- Need for antiglaucoma medication in addition to surgery.

## Results

**Base line characteristics**

The table below depicts the baseline characteristics of patients in the two groups (**Table 1**).

**Tabel 1:** The baseline characteristics of patients in the two groups

BASELINE CHARACTERISTICS	CONVENTIONAL	RELESABLE
AGE	55 ± 10.48 yrs	59 ± 12.42 yrs.
ANTIGLAUCOMA MEDICATION	3 ± 1.0	3 ± 1.0
PRE-OP IOP	33 ± 12	39 ± 13
POST OP IOP	12.8 ± 4.0	9.0 ± 3.6

**Intraocular pressure**

The bar below shows changes in intraocular pressure during follow-up for both groups. The mean preoperative intraocular pressure was 33 ± 12 mmHg in the single suture group and 39 ± 13 mmHg in the releasable suture group (p). We observed a highly significant reduction of intraocular pressure at all times in both groups compared with the preoperative intraocular pressure (P, 0.0001). Intraocular pressure was 12.8 ± 4.0 mmHg in the single suture group and 9.0 ± 3.6 mmHg in patients with releasable sutures at the last follow-up.

Removal of releasable sutures was performed in the event of inadequate filtration and unacceptable intraocular pressure. Some patients in the releasable suture group needed release of sutures, most often during the first week after trabeculectomy (range 1–27 days). 

**Fig. 1:**
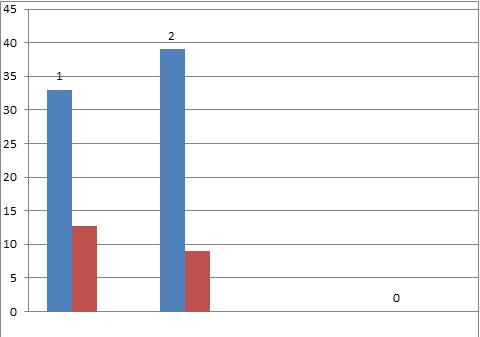
Releasable suture in a case of releasable suture trabeculectomy

Removal of one releasable suture was performed under slit-lamp with topical anesthesia, and second suture was released within three weeks (**Fig. 1**).

**Fig. 2:**
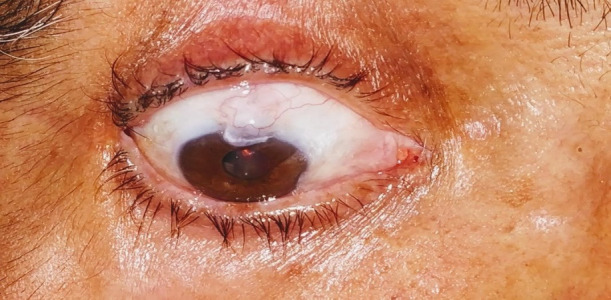
Average pre- and post-operative intraocular pressure in patients in the two groups

**Fig. 2** shows pre op and post op IOPs in conventional and releasable suture trabeculectomy respectively [**1**,**2**].

A comparison of bleb morphology using Indiana Bleb Classification is shown in **Table 2**. 

**Tabel 2:** Comparison of bleb morphology using Indiana Bleb Classification

	CONVENTONAL	RELESABLE	P VALUE
BLEB HEIGHT	2.25 (1.75-2.5)	1.75 (1.5-2)	SIGNIFICANT
HORIZONTAL EXTENT	2.23 (2.15-2.75)	2.75 (2.50-3.15)	SIGNIFICANT
VASCULARITY	2.10 (1.90-2.75)	1.65 (1.23-1.90)	SIGNIFICANT
LEAK	0.15 (0.04-0.45)	0.08 (0.02-0.1)	INSIGNIFICANT

**Fig. 3:**
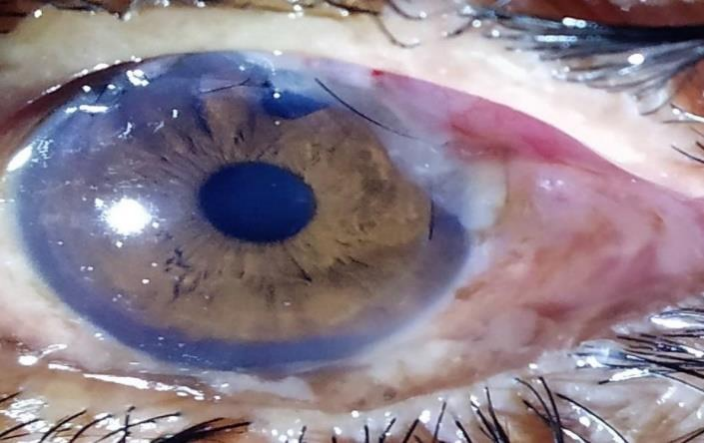
Low lying, diffuse, and more ideal vascularity bleb

There was an obvious difference between the bleb morphology between conventional trabeculectomy and releasable suture trabeculectomy. Blebs in releasable suture trabeculectomy were more diffuse, low lying and presented a more ideal vascularity (**Fig. 3**).

**Complications**

Shallow AC and bleb leak were looked for in both groups in the immediate postoperative period. Shallow AC was noticed in 8 patients in conventional trabeculectomy and in three patients in releasable suture trabeculectomy. Complicated cataract was noticed in 2 patients in conventional trabeculectomy and in none of the patients in releasable suture trabeculectomy.

**Medication**

Pre operatively, 56% of the patients with conventional trabeculectomy were on treatment with 3 ± 1 medications and 69% of the patients with releasable suture were on 3 ± 1.5 medications. At the end of 12 months follow up period, 35% of the patients with conventional trabeculectomy and 20% of the patients with releasable suture trabeculectomy were on one or two medications until one year follow up. However, the difference was not statistically significant.

## Discussion

Trabeculectomy without releasable sutures has already been documented to be associated with a significantly higher frequency of hypotony and a flat anterior chamber than trabeculectomy with releasable sutures [**9**]. Very few studies have been done to show the influence of releasable sutures in trabeculectomy. In our study, we found releasable suture trabeculectomy to be an extremely useful technique. IOP lowering effect was much more present as compared to conventional trabeculectomy. The releasable suture trabeculectomy was introduced mainly to overcome possible complications like hypotony, a shallow flat anterior chamber and choroidal detachment. IOP assessment could be done and releasable suture can be removed as per IOP response. Though IOP was reduced with both procedures, the drop in IOP was numerically more in releasable suture trabeculectomy and significant too. Our study was supported by a similar study by Thomas R et al. [**10**]. Releasable suture trabeculectomy also decreases the complications during early postoperative period [**11**]. Many previous trial studies [**12**-**14**] have documented the efficacy and safety of trabeculectomies with or without releasable sutures. Releasable suture trabeculectomy has well been documented to reduce the complications as compared to conventional trabeculectomy [**9**]. The lower incidence of complications is because of the tight suturing of the flap. In our study, we found that the results in terms of IOP lowering were comparable and complications were greatly reduced in the releasable suture group. Both these findings were consistent with meta-analysis of Zhon et al. [**9**]. There was a significant reduction in the use of antiglaucoma medication postoperatively in both groups. Our study has come up with the assumption that since the releasable sutures are removed in a couple of weeks, they may possibly play a role in wound modulation [**15**-**17**] and formation of a more diffuse bleb. However, this assumption needs more evaluation on a larger sample. But what we observed in our study, the IOP control, has been excellent and bleb has been more diffuse and mildly elevated.

An incomplete wound healing at the site of filtering surgery is necessary, which is against most other surgeries that complete healing and restoration of normal architecture [**18**]. Early postoperative period is the most important phase and interventions should be performed at this stage to prevent any arising failure [**19**]. According to Savage et al., releasable sutures are removable sutures, their removal in early postoperative period can adjust the filtration flow in a controlled fashion [**20**]. Our study supports the similar proposition too. Shingleton [**21**] correlated morphological features found in blebs to the level of IOP. Thus, we can correlate this with our study and suggest that releasable sutures may possibly affect the bleb formation and the result is usually a favorable one.

We believe that removal of sutures also helped in achieving a good mildly elevated bleb. Besides the fact that none of our patients presented with any complicated cataract, we cannot deny that trabeculectomy hastens the cataract formation in phakic patients. The overall IOP was in many patients in single digits. In addition, shallow AC was seen in 3 patients and in immediate post-operative patients and formed over a period of 2-3 days with conservative measures.

## Conclusion

Releasable suture trabeculectomy is a far better technique than conventional trabeculectomy. Results are very good in terms of IOP control, post-operative complications, and bleb morphology. They may possibly have a role in wound modulation, thereby achieving an ideal bleb, though many large sample studies still need to be done. Such technique needs to be encouraged when dealing with the surgical patients in our day-to-day practice.

**Conflict of Interest**

The authors declare no conflict of interest.

**Informed Consent and Human and Animal Rights statements**

Informed consent has been obtained from all individuals included in this study.

**Authorization for the use of human subjects**

Ethical approval: The research related to human use complies with all the relevant national regulations, institutional policies, is in accordance with the tenets of the Helsinki Declaration, and has been approved by the Institutional Ethics Committee.

**Acknowledgements**

None.

**Sources of Funding**

None.

**Disclosures**

None. 
